# Nephrotoxic Effects of Endocrine-Disrupting Chemicals: A Systematic Review

**DOI:** 10.7759/cureus.99603

**Published:** 2025-12-19

**Authors:** Anas E Ahmed, Manal H Madkhali, Hashim A Alhashim, Laura M Damanhouri, Ayman M Bagasher, Abdulmajeed M Alharbi, Ayman F Almohammadi, Nadia A Alomrani, Abdulkarim M Alghaithi, Nouf H Hibili

**Affiliations:** 1 Community Medicine, Jazan University, Jazan, SAU; 2 Faculty of Medicine, Jazan University, Jazan, SAU; 3 General Practice, Al-Ahsa Health Cluster, Al-Hofouf, SAU; 4 College of Medicine and Surgery, King Abdulaziz University, Jeddah, SAU; 5 College of Medicine, Hail University, Hail, SAU; 6 Faculty of Medicine, King Abdulaziz University, Rabigh, SAU; 7 General Practice, Tabuk Health Cluster, Tabuk, SAU; 8 Faculty of Medicine, Umm Al-Qura University, Makkah, SAU

**Keywords:** apoptosis, bisphenols, chronic kidney disease, endocrine-disrupting chemicals, heavy metals, nephrotoxicity, oxidative stress, phthalates, renal dysfunction, volatile organic compounds

## Abstract

Endocrine-disrupting chemicals (EDCs) are widespread environmental pollutants that interfere with hormonal regulation and are increasingly linked to kidney dysfunction. The kidney’s central role in xenobiotic filtration and excretion makes it vulnerable to cumulative toxic exposures that trigger oxidative stress, inflammation, and apoptosis, leading to chronic kidney disease (CKD) and related disorders. This systematic review synthesized evidence from human, animal, and computational studies assessing exposure to multiple EDC classes, including bisphenols, phthalates, parabens, alkylphenols, volatile organic compounds (VOCs), per- and polyfluoroalkyl substances (PFASs), pesticides, and heavy metals, and their associations with kidney outcomes. A comprehensive search of PubMed, Scopus, Web of Science, and the Cochrane Library was conducted up to October 2025 following Preferred Reporting Items for Systematic Reviews and Meta-Analyses (PRISMA) guidelines. Methodological quality was evaluated using the AXIS tool for cross-sectional studies, the Newcastle-Ottawa Scale (NOS) for cohort and case-control studies, the Joanna Briggs Institute (JBI) checklists, and the Toxicological Data Reliability Assessment Tool (ToxRTool). Of 42,006 records screened, 10 studies met the inclusion criteria, encompassing large population cohorts, animal experiments, and molecular docking analyses. Cross-sectional datasets, such as the National Health and Nutrition Examination Survey (NHANES), demonstrated significant associations between VOCs, phthalates, and heavy metals (particularly lead and cadmium) and increased CKD and diabetic kidney disease (DKD) risk, while some PFASs showed neutral or inverse associations. Experimental evidence confirmed that bisphenol and alkylphenol derivatives induce renal injury via oxidative stress, p53 activation, and apoptosis, and dialysis-related studies revealed iatrogenic bisphenol contamination. Overall, these findings highlight the kidney’s susceptibility to environmental toxicants and emphasize the need for stricter regulation of EDC-containing materials, improved biomonitoring of cumulative exposures, and further mechanistic research to clarify dose-response relationships and long-term renal effects.

## Introduction and background

Endocrine-disrupting chemicals (EDCs) are exogenous compounds that interfere with normal hormone regulation and disrupt endocrine function. These chemicals include bisphenols, phthalates, parabens, alkylphenols, per- and polyfluoroalkyl substances (PFASs), volatile organic compounds (VOCs), and certain heavy metals [1-3]. Because they are prevalent in consumer products and industrial processes, human exposure is widespread and often persistent [4-6].

Exposure occurs through contaminated food and water, polluted air, and contact with everyday products [7,8]. Persistent EDCs, such as PFASs and heavy metals, can accumulate in tissues, and measurable levels are frequently detected in human samples [9,10]. Vulnerable groups, including pregnant women, children, and individuals with chronic illnesses, may be more sensitive to these chemicals’ effects [6]. Although some EDCs have been restricted, newer substitutes continue to demonstrate similar toxic properties [1,2,5].

The kidney, a key organ responsible for filtration and excretion, is particularly susceptible to EDC-related injury [4,5,7]. Experimental studies show that many EDCs trigger oxidative stress, inflammation, mitochondrial dysfunction, and cell death in renal tissues [1,3,5]. These effects reflect chemical-specific disruptions in metabolic, endocrine, and immune signaling pathways [1-3].

Epidemiological studies have linked exposure to various EDCs, including phthalates, bisphenols, VOCs, PFASs, and heavy metals, to markers of reduced kidney function, such as elevated albumin-to-creatinine ratio (ACR) and decreased estimated glomerular filtration rate (eGFR), as well as clinical outcomes such as chronic kidney disease (CKD) and diabetic kidney disease (DKD) [2-4,7-10]. However, heterogeneity in study design and exposure assessment has contributed to inconsistent findings [1,5].

Given the increasing burden of CKD, understanding environmental contributors is essential. This systematic review aims to synthesize evidence across multiple EDC classes and kidney outcomes, clarify mechanisms of nephrotoxicity, and highlight gaps to guide future research and regulatory efforts.

## Review

Methodology

Literature Search Strategy

This systematic review adhered to the Preferred Reporting Items for Systematic Reviews and Meta-Analyses (PRISMA 2020) guidelines [11]. A comprehensive search of PubMed, Scopus, Web of Science, and the Cochrane Library was performed from database inception to October 2025 to identify observational, experimental, and mechanistic studies examining associations between EDC exposure and kidney-related outcomes.

Search strategies combined subject headings and free-text terms for major EDC classes (bisphenols, phthalates, parabens, alkylphenols, PFASs, VOCs, heavy metals, pesticides) and renal outcomes, including CKD, DKD, renal dysfunction, and nephrotoxicity. Searches were limited to English-language human studies. Reference lists of included studies and relevant reviews were also screened.

Eligibility Criteria

Eligibility followed the Population, Exposure, Comparator, Outcome (PECO) framework [12]. Studies involving humans of any age with measured or estimated exposure to one or more EDCs were included. Comparators were groups with lower or no exposure.

Eligible outcomes included kidney biomarkers (serum creatinine, eGFR, ACR), clinical diagnoses (CKD, DKD), renal transplant outcomes, and mechanistic indicators of nephrotoxicity (oxidative stress, inflammation, apoptosis). Observational designs, randomized trials, and mechanistic experiments were eligible.

Exclusion criteria included studies lacking quantitative exposure or kidney outcomes, non-human research without clear translational relevance, reviews, conference abstracts, editorials, non-English publications, and non-peer-reviewed material.

Study Selection

**Figure 1:**
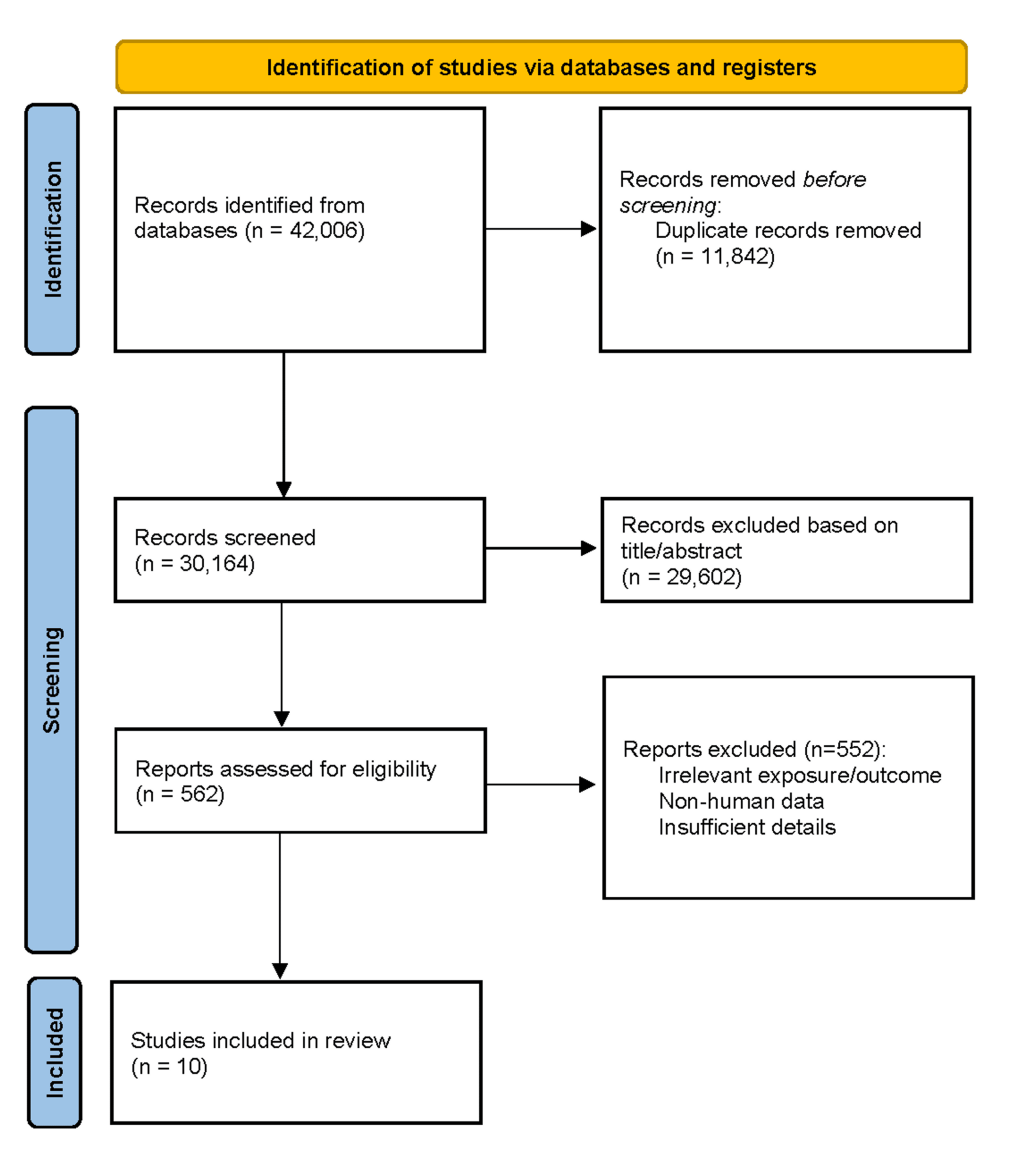
PRISMA flow diagram of the study selection process The Preferred Reporting Items for Systematic Reviews and Meta-Analyses (PRISMA) flow diagram illustrates the process of study identification, screening, eligibility assessment, and inclusion in the review in accordance with PRISMA guidelines [11]. Records were identified through database searching and other sources, duplicates were removed, and the remaining records were screened based on titles and abstracts. Full-text articles were assessed for eligibility, with reasons for exclusion documented. Studies meeting the predefined inclusion criteria were included in the final qualitative and/or quantitative synthesis.

Two reviewers independently screened all records through title and abstract review, followed by full-text assessment. Disagreements were resolved by discussion or a third reviewer. The PRISMA flow diagram details the number of records identified, screened, excluded, and included (Figure 1).

Data Extraction and Quality Appraisal

Data were independently extracted by two reviewers using a standardized template, recording study characteristics, population details, exposure assessment methods, kidney outcomes, and key findings, including effect estimates and mechanistic results.

Quality appraisal used established tools: the AXIS tool for cross-sectional studies [13], the Newcastle-Ottawa Scale (NOS) for cohort and case-control studies [14], and the SYstematic Review Center for Laboratory animal Experimentation (SYRCLE) tool for mechanistic or animal studies [15]. Studies were rated as low, moderate, or high risk of bias. Only studies meeting quality thresholds were included in the synthesis, with findings organized by EDC class and renal outcome.

Results

Study Selection

A total of 42,006 records were identified across PubMed, Scopus, Web of Science, and the Cochrane Library. After removing duplicates, 30,164 unique studies were screened by title and abstract, excluding 29,602 irrelevant records. Full texts of 562 articles were assessed, with 552 excluded due to irrelevant exposure or outcome, non-human data, or insufficient methodology. Ten studies met the inclusion criteria for qualitative synthesis, while none were suitable for meta-analysis due to heterogeneity in design, populations, and outcome measures [1-10]. The PRISMA flow diagram summarizes the identification, screening, and selection process (Figure 1).

Study Characteristics

The 10 included studies encompassed diverse methodologies and geographic regions, integrating epidemiological, experimental, and computational approaches to investigate the relationship between EDCs and kidney outcomes. Li et al. analyzed adults with diabetes from the National Health and Nutrition Examination Survey (NHANES), assessing metals, PFASs, and VOCs [2]. Ma et al. employed in vivo murine models and in vitro HK-2 cell systems to investigate Bisphenol P (BPP) nephrotoxicity, while Pei et al. used in silico network toxicology to explore nonylphenol (NP) and octylphenol (OP) interactions with CKD-related targets [1,5]. Other studies examined urinary PAH biomarkers in a Chinese cohort [9], BPA and chlorinated derivatives in ESRD patients [4], multi-pollutant exposure in healthy Korean women [8], and dialysis fluid contamination [7]. Additional research addressed maternal occupational exposure and congenital kidney anomalies [6], occupational VOC exposure among Nigerian workers [6], and longitudinal PFAS exposure in the Fernald Community Cohort [3]. Exposure assessments included high-performance liquid chromatography-mass spectrometry (HPLC-MS)/MS, job-exposure matrices, and serum analyses, while kidney outcomes ranged from biochemical markers to developmental and histopathological endpoints. Together, these studies highlight the multifaceted nature of EDC exposure and nephrotoxicity research (Table 1).

**Table 1:** Summary of the characteristics of the included studies assessing the impact of endocrine-disrupting chemicals (EDCs) on renal outcomes in human populations, animal models, and in silico systems Renal outcomes include diabetic kidney disease (DKD), chronic kidney disease (CKD), estimated glomerular filtration rate (eGFR), and serum or urinary renal markers. Exposure to EDCs, such as metals (Pb, Cd, Hg, Se, Mn), per- and polyfluoroalkyl substances (PFAS), bisphenols, phthalates, benzophenones, polycyclic aromatic hydrocarbons (PAHs), volatile organic compounds (VOCs), and alkylphenols, was assessed via biological samples or occupational/job-exposure matrices. Statistical approaches included regression models, weighted quantile sum (WQS) analyses, Bayesian kernel machine regression (BKMR), and molecular docking/network analyses. ACR – albumin-to-creatinine ratio; BPA – bisphenol A; ClxBPA – chlorinated BPA derivatives; BPP – bisphenol P; CAKUT – congenital anomalies of the kidney and urinary tract; CREA – serum creatinine; DEHP – di(2-ethylhexyl) phthalate; DKD – diabetic kidney disease; eGFR – estimated glomerular filtration rate; HD – hemodialysis; HDF – hemodiafiltration; MBP – mono-n-butyl phthalate; MiBP – mono-isobutyl phthalate; MBzP – monobenzyl phthalate; NAC – N-acetylcysteine; NP – nonylphenol; OP – octylphenol; PAH – polycyclic aromatic hydrocarbon; PFAS – per- and polyfluoroalkyl substances; ROC – receiver operating characteristic; RCS – restricted cubic spline; UACR – urinary albumin-to-creatinine ratio; UA – uric acid; UREA – serum urea; VOC – volatile organic compound; WQS – weighted quantile sum regression; NHANES – National Health and Nutrition Examination Survey

Author	Country/Population	Study Design	Sample Size	Type of EDC(s)	Exposure Assessment	Renal Outcome(s)	Statistical Analysis	Main Findings
Pei et al. [1]	China – computational toxicology datasets	Network toxicology + molecular docking	In silico	Nonylphenol (NP) & Octylphenol (OP)	Targets from CTD + GeneCards; docking & MD simulations with ESR1, MAPK3, TNF, BCL2, FOS	CKD pathogenesis + diagnostic modeling	Network + enrichment (GO/KEGG); logistic regression; ROC; molecular docking & dynamics	NP & OP linked to oxidative stress, apoptosis, immune pathways; hub genes (TNF, MAPK3, ESR1, FOS, BCL2) implicated; strong binding energies ( 0.7.
Li et al. [2]	USA – NHANES 2015–2018 adults with diabetes	Cross-sectional (national survey)	1,421 participants (575 DKD, 846 non-DKD)	Metals (Pb, Cd, Hg, Se, Mn); PFASs (PFHxS, PFNA, n-PFOA, n-PFOS, Sm-PFOS); VOC metabolites (2HPMA, MHBMA3, PGA)	Urine, blood, and serum measured by HPLC–MS/MS	Diabetic kidney disease (albuminuria ≥30 mg/g or eGFR ≤ 60 mL/min/1.73 m²)	Logistic regression, restricted cubic spline (RCS), Weighted Quantile Sum (WQS), Bayesian Kernel Machine Regression (BKMR), mediation analysis	Pb (OR = 1.76, 95% CI 1.13–2.74) and certain VOCs positively associated with DKD; PFASs inversely related. Co-exposure (metals + metalloids) increased DKD risk (WQS OR = 2.34). Pb effect partly mediated by serum globulins (7.25%).
Blake et al. [3]	USA – Fernald Community Cohort (adults exposed to PFAS)	Longitudinal (18-year follow-up)	210 participants	PFAS (PFOA, PFOS, PFNA, PFHxS, PFDeA, PFOSA, Me-PFOSA, Et-PFOSA)	Serum HPLC–MS/MS (1991–2008)	eGFR (CKD-EPI)	Linear mixed-effects models (adjusted for demographics)	PFNA, PFHxS, PFDeA → ↓ eGFR (−1.61 to −2.20%, p < 0.05); PFOS, PFOSA negative trend; Me-PFOSA positive (+1.53%); suggests chronic PFAS exposure reduces renal filtration capacity.
Cambien et al. [4]	France – ESRD patients on dialysis & CKD5 patients vs controls	Observational (pre-/post-dialysis)	64 ESKDD (32 HD, 32 HDF), 36 CKD5, 24 controls	Bisphenol A (BPA) & chlorinated derivatives (ClxBPAs: MCBPA, DCBPA, TCBPA, TTCBPA)	Plasma UHPLC–MS/MS before & after dialysis	Plasma BPA & ClxBPA levels; renal markers	t-test, Mann–Whitney, Wilcoxon, Kendall correlation, χ²	BPA 22.5× higher in ESKDD vs controls (2.21 vs 0.10 ng/mL); dialysis failed to reduce levels — sometimes increased; polysulfone dialyzers raised BPA exposure; ClxBPAs detected in ~38% patients; cumulative toxicity likely.
Ma et al. [5]	China – C57BL/6 mice + HK-2 renal cells	Experimental (in vivo + in vitro)	24 mice (4×6) + cell assays	Bisphenol P (BPP)	Oral gavage (0.3–3000 µg/kg bw/day, 5 weeks); HK-2 cells 0–30 µmol/L BPP	Serum renal markers (UA, UREA, CREA); renal histopathology; oxidative stress + apoptosis indices	One-way ANOVA; RNA-seq (DESeq2); KEGG/GO/GSEA; qRT-PCR; Western blot	BPP caused renal dysfunction and histologic injury; ↑ UA, UREA, CREA; oxidative stress and p53-mediated apoptosis implicated; NAC attenuated injury, confirming oxidative-stress mechanism.
Spinder et al. [6]	Netherlands – Mothers & offspring (EUROCAT NNL & Lifelines cohorts)	Case–control (registry + population)	530 CAKUT, 364 hypospadias, 5602 controls	Occupational EDCs (organic solvents, alkylphenols, phthalates, benzophenones, parabens, siloxanes, PAHs, pesticides)	Job-exposure matrix linked to ISCO88 codes	CAKUT and hypospadias in offspring	Logistic regression (adjusted for maternal & perinatal covariates)	Organic solvents/alkylphenols (aOR 1.41) and phthalates/benzophenones/parabens/siloxanes (aOR 1.56) linked to CAKUT; strongest for urinary collecting system (aOR 1.62); no hypospadias association.
Bacle et al. [7]	France – ESRD patients undergoing online hemodiafiltration (OL-HDF)	Observational (lab + clinical)	Samples from dialysis systems and fluids (no patients enrolled)	BPA and chlorinated derivatives (MCBPA, DCBPA, TCBPA, TTCBPA)	Measured in dialysis water, dialysate, replacement fluid (SPE–UPLC–MS/MS)	BPA & ClxBPA levels in fluids (ESRD exposure risk)	Wilcoxon, Friedman post-hoc (p < 0.05)	BPA and ClxBPA leached from dialyzers and water systems; dialysate BPA ↑ 5×, replacement fluid ↑ 50× (1033 ng/L); ClxBPA (esp. DCBPA) detected; indicates high iatrogenic BPA exposure risk.
Kang et al. [8]	South Korea – healthy adult females (20–45 yrs)	Cross-sectional (multi-pollutant)	441 participants	Phthalates (MBP, MiBP, MBzP, DEHP) and Benzophenones (BP-1, BP-3)	Urinary concentrations adjusted for creatinine & SG (LC–MS/MS)	Urinary albumin-to-creatinine ratio (ACR)	Linear regression (single & multi-pollutant), sensitivity analysis	MBP (β = 0.15) and BP-1 (β = 0.11) associated with ↑ ACR (p < 0.001); significant after adjustment; DEHP & BPA not significant; suggest DBP & BP metabolites as potential nephrotoxic EDCs.
Sachs-Strømmen et al. [9]	China – NHANES 2009–2018 adults	Cross-sectional (population-based)	22,539 participants	Polycyclic aromatic hydrocarbons (PAHs)	Urinary 1-, 2-, 3-, 9-hydroxyphenanthrene; 1-hydroxypyrene (HPLC–MS/MS)	eGFR (CKD-EPI), UACR, CKD prevalence	Weighted logistic regression, RCS, WQS, BKMR	Higher urinary PAHs associated with ↓ eGFR and ↑ CKD risk (WQS OR = 1.21, 95% CI 1.03–1.42); nonlinear exposure–response noted; 1-hydroxypyrene main driver; synergistic nephrotoxicity via oxidative stress & inflammation.
Mu et al. [10]	Nigeria – petrol station workers vs controls	Cross-sectional (human occupational)	60 (30 exposed, 30 controls)	VOCs (benzene, toluene, xylene)	Occupational inhalation (petrol vapors); blood biochemistry	Serum creatinine, urea, uric acid (+ hepatic markers)	t-test, ANOVA (p < 0.05)	Exposed group showed ↑ creatinine, urea, uric acid (p < 0.05); renal and hepatic dysfunction linked to chronic VOC exposure — suggests petroleum EDCs cause nephrotoxicity.

Quality Assessment

Cross-sectional studies were evaluated using the AXIS tool, with four of five studies showing low risk of bias [2,8-10]. One study exhibited moderate-to-high risk due to a small convenience sample, indirect occupational exposure estimation, and lack of confounder adjustment [10]. Experimental and quasi-experimental studies were appraised using JBI checklists, while case-control and cohort studies were assessed using the NOS. Ma et al. and Pei et al. were considered low risk, while Cambien et al. and Bacle et al. were rated low-to-moderate risk due to limited confounder control [1,4,5,7]. Spinder et al. and Blake et al. were judged low risk, and the in silico study by Pei et al. was further evaluated using ToxRTool, confirming low bias across measured domains [1,3,6,16]. Overall, the included studies demonstrated adequate methodological quality, though some limitations in exposure assessment and confounder adjustment were noted (Table 2).

**Table 2:** Summary of the quality appraisal of the included studies Risk-of-bias assessment of studies evaluating the effects of endocrine-disrupting chemicals (EDCs) on renal outcomes. The assessment was based on standardized tools: AXIS – Appraisal tool for Cross-Sectional Studies [13] and NOS – Newcastle–Ottawa Scale for cohort and case–control studies [14]. The overall risk of bias was classified as low, low–moderate, or moderate based on study design, sample size, exposure/outcome measurement, confounder control, and analytical rigor. Concise justifications highlight key strengths and limitations of each study. NHANES – National Health and Nutrition Examination Survey

Study (Author)	Study Design	Tool Used	Overall Risk of Bias	Concise Justification
Pei et al. [1]	In silico network toxicology	ToxRTool	Low risk	Transparent data sources, validated software, reproducible workflow; limitation: lacks experimental validation.
Li et al. [2]	Cross-sectional (NHANES, USA)	AXIS	Low risk	Nationally representative design, standardized biomarker assays, full confounder adjustment; minor limitation: no power analysis or handling of non-response.
Blake et al. [3]	Longitudinal cohort (Fernald PFAS cohort, USA)	NOS	Low risk	18-year repeated PFAS measures, strong confounder control, advanced mixed-effects modeling; minimal attrition bias.
Cambien et al. [4]	Analytical cross-sectional (CKD5, ESRD)	JBI Cross-sectional	Low–moderate risk	Highly reliable UHPLC–MS/MS quantification, but no regression adjustment for confounders (e.g., diet, comorbidities).
Ma et al. [5]	Experimental (mice + HK-2 cells)	JBI Experimental	Low risk	Randomized groups, validated exposures and outcomes, comprehensive mechanistic analyses; minor limitation: unclear blinding, small group size.
Spinder et al. [6]	Case–control (maternal EDC exposure & CAKUT)	NOS	Low risk	Registry-based design, blinded JEM exposure coding, multivariate adjustment; limitation: lacks quantitative exposure data.
Bacle et al. [7]	Quasi-experimental (dialysis exposure study)	JBI Quasi-experimental	Low–moderate risk	Excellent analytical QA/QC, triplicate measures, but confounders not modeled statistically.
Kang et al. [8]	Cross-sectional (Korean women)	AXIS	Low risk	Validated LC–MS/MS analysis, strong statistical modeling, well-controlled confounders; minor limitation: no non-responder analysis.
Sachs-Strømmen et al. [9]	Cross-sectional (NHANES, China)	AXIS	Low risk	Large sample, rigorous exposure and renal function measurements, appropriate modeling (WQS, BKMR); limitation: cross-sectional temporality.
Mu et al. [10]	Cross-sectional (NHANES, USA)	AXIS	Low risk	Nationally weighted dataset, 28 EDCs measured with LC–MS/MS, advanced quantile regression; limitation: no power calculation.

Specific Findings Across Chemical Classes

Evidence across the included studies demonstrates that multiple classes of EDCs exert measurable effects on renal structure and function through diverse toxicological mechanisms. VOCs and heavy metals showed consistent associations with adverse renal outcomes in population datasets. In adults with diabetes from NHANES, Li et al. reported that urinary VOC metabolites, such as 2-hydroxypropyl mercapturic acid (2HPMA), monohydroxybutenyl mercapturic acid (MHBMA3), and phenylglyoxylic acid (PGA), along with blood lead (Pb) concentrations, were associated with a higher probability of diabetic kidney disease (DKD), while certain per- and polyfluoroalkyl substances (PFASs), including perfluorohexanesulfonic acid (PFHxS) and perfluorooctanoic acid (n-PFOA), demonstrated inverse associations [2]. Mixture modeling using weighted quantile sum (WQS) regression and Bayesian kernel machine regression (BKMR) identified Pb and MHBMA3 as dominant contributors, and the authors observed effect modification by sex and metabolic factors. These findings suggest that metals and VOCs may contribute to renal impairment primarily through oxidative and inflammatory pathways.

Experimental and mechanistic analyses strengthen the evidence for nephrotoxicity among bisphenol derivatives. Ma et al. demonstrated in both mice and HK-2 human renal proximal tubular cells that bisphenol P (BPP) exposure results in dose-dependent renal dysfunction, characterized by increased serum creatinine, urea, and uric acid, along with tubular epithelial detachment, inflammatory infiltration, mitochondrial dysfunction, and apoptosis [5]. Transcriptomic profiling revealed differentially expressed genes enriched in the p53 signaling pathway and oxidative stress responses, and antioxidant pretreatment reversed many of these effects, confirming oxidative stress as a central mechanism. Complementing these findings, Pei et al. conducted an in silico systems-toxicology assessment of NP and OP [1]. Their analysis identified 49 overlapping genes between chemical targets and chronic kidney disease (CKD)-related pathways, with enrichment of tumor necrosis factor (TNF), interleukin-17 (IL-17), mitogen-activated protein kinase (MAPK), and NOD-like receptor signaling, and molecular docking confirmed strong binding to MAPK3 and TNF. Together, these studies indicate that alkylphenols and bisphenols interact with key inflammatory and apoptotic pathways relevant to renal injury.

Phthalate and phenolic exposures were also implicated in renal alterations in both pediatric and adult populations. In a cohort of Norwegian pediatric kidney transplant recipients, Sachs-Strømmen et al. found universal detection of di(2-ethylhexyl) phthalate (DEHP), diisononyl phthalate (DiNP), bisphenol A (BPA), parabens, and triclosan, often exceeding European thresholds for BPA safety [9]. Girls and children with metabolic syndrome exhibited higher levels of monoethyl phthalate (MEP), which was positively correlated with estimated glomerular filtration rate (eGFR), raising concerns about heightened susceptibility to environmental toxicants in post-transplant physiology. In healthy Korean women, Kang et al. observed that several phthalate metabolites (particularly mono-n-butyl phthalate (MBP)) and benzophenones (especially BP-1) were associated with higher urinary albumin-to-creatinine ratios (ACR), suggesting early glomerular dysfunction [8]. A broader evaluation of multi-chemical exposures in NHANES by Mu et al. demonstrated that combined burdens of phthalates, phenols, PFASs, and metals were associated with lower eGFR and increased CKD prevalence, with DEHP metabolites, parabens, and Pb contributing most strongly [10].

Patients with advanced kidney disease appear to face unique risks related to bisphenol exposure. Cambien et al. showed that BPA concentrations in end-stage renal disease (ESRD) and CKD stage 5 (CKD5) patients were substantially higher than in healthy controls and that dialysis sessions failed to reduce circulating levels [4]. Approximately one-third of patients had detectable chlorinated BPA derivatives (ClxBPA). Bacle et al. traced these elevations to contamination originating from dialysis water, dialyzers, and replacement fluids, leading to significant iatrogenic exposure [7]. These findings highlight the vulnerability of dialysis-dependent individuals to device-related chemical leaching.

Maternal occupational exposure also emerged as an important determinant of renal developmental outcomes. Spinder et al. reported that prenatal exposure to mixtures of organic solvents, alkylphenols, phthalates, parabens, benzophenones, and siloxanes was associated with congenital anomalies of the kidney and urinary tract (CAKUT), with the strongest associations observed for abnormalities of the urinary collecting system [6]. Sex-specific differences suggested possible endocrine-mediated susceptibility during fetal development.

Findings for PFAS exposure were more heterogeneous. In a longitudinal 18-year evaluation, Blake et al. found that perfluorooctanesulfonic acid (PFOS) and n-PFOA were associated with altered thyroid hormone levels but did not consistently predict eGFR decline [3]. These results indicate potential endocrine interactions without clear long-term renal effects in this cohort.

## Conclusions

This systematic review demonstrates that exposure to endocrine-disrupting chemicals, including bisphenols, phthalates, VOCs, PFASs, and heavy metals, negatively impacts kidney function across the lifespan. These effects involve oxidative stress, apoptosis, inflammatory signaling, and disruption of hormonal pathways. Vulnerable populations include individuals with preexisting kidney disease, dialysis patients, and developing fetuses. The widespread presence and persistence of these chemicals highlight the need for regulatory measures, clinical monitoring, and further mechanistic research to mitigate long-term renal and systemic health risks.
